# Microorganisms Involved in Methylmercury Demethylation and Mercury Reduction are Widely Distributed and Active in the Bathypelagic Deep Ocean Waters

**DOI:** 10.1021/acs.est.4c00663

**Published:** 2024-07-24

**Authors:** Isabel Sanz-Sáez, Andrea G. Bravo, Marta Ferri, Joan-Martí Carreras, Olga Sánchez, Marta Sebastian, Clara Ruiz-González, Eric Capo, Carlos M. Duarte, Josep M. Gasol, Pablo Sánchez, Silvia G. Acinas

**Affiliations:** † Departament de Biologia Marina i Oceanografia, Institut de Ciències del Mar, ICM-CSIC, 08003 Barcelona, Catalunya, Spain; ‡ Departament de Genètica i Microbiologia, Facultat de Biociències, Universitat Autònoma de Barcelona (UAB), 08193 Bellaterra, Spain; § Red Sea Research Center, Division of Biological and Environmental Sciences and Engineering, King Abdullah University of Science and Technology, Thuwal 23955-6900,Saudi Arabia

**Keywords:** mercury, methylmercury, bathypelagic, bacterial demethylation, metagenomes, metatranscriptomes, *mer* genes

## Abstract

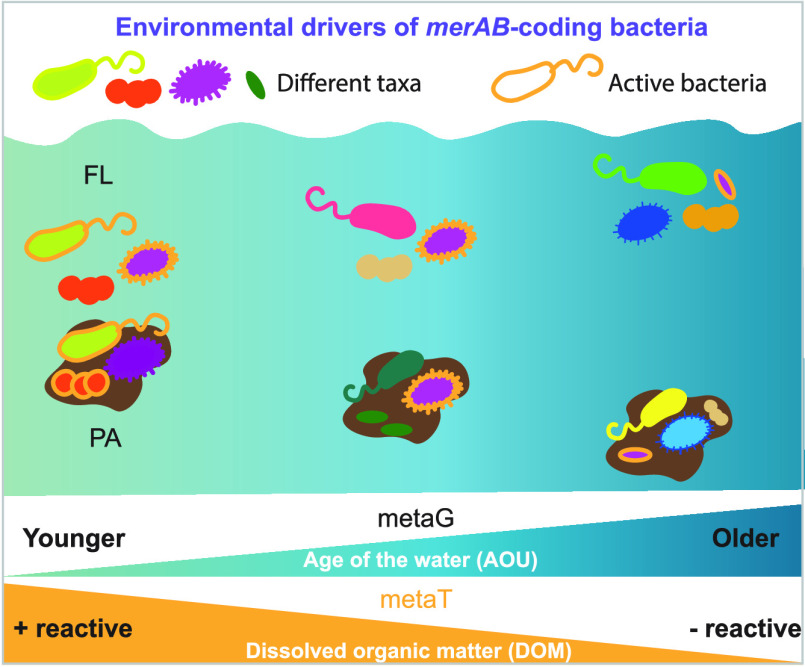

The ocean’s mercury (Hg) content has tripled due to anthropogenic activities, and although the dark ocean (>200 m) has become an important Hg reservoir, concentrations of the toxic and bioaccumulative methylmercury (MeHg) are low and therefore very difficult to measure. As a consequence, the current understanding of the Hg cycle in the deep ocean is severely data-limited, and the factors controlling MeHg, as well as its transformation rates, remain largely unknown. By analyzing 52 globally distributed bathypelagic deep-ocean metagenomes and 26 new metatranscriptomes from the Malaspina Expedition, our study reveals the widespread distribution and expression of bacterial-coding genes *merA* and *merB* in the global bathypelagic ocean (∼4000 m depth). These genes, associated with Hg^II^ reduction and MeHg demethylation, respectively, are particularly prevalent within the particle-attached fraction. Moreover, our results indicate that water mass age and the organic matter composition shaped the structure of the communities harboring *merA* and *merB* genes living in different particle size fractions, their abundance, and their expression levels. Members of the orders *Corynebacteriales*, *Rhodobacterales*, *Alteromonadales*, *Oceanospirillales*, *Moraxellales*, and *Flavobacteriales* were the main taxonomic players containing *merA* and *merB* genes in the deep ocean. These findings, together with our previous results of pure culture isolates of the deep bathypelagic ocean possessing the metabolic capacity to degrade MeHg, indicated that both methylmercury demethylation and Hg^II^ reduction likely occur in the global dark ocean, the largest biome in the biosphere.

## Abstract 2

Methylmercury (MeHg) accumulates in aquatic food webs, posing a human health risk through fish consumption. Despite being present globally, measuring MeHg in oceans remains challenging, hindering understanding of its formation and degradation. Our study proposes the *mer* operon’s role in MeHg demethylation, aiming to analyze key genes (*merA* and *merB*) to gain insights into the biogeochemical cycle of Hg in the deep ocean.

## Introduction

Concentrations of mercury (Hg) in the Earth’s atmosphere, oceans, and soils have tripled during the last two centuries due to anthropogenic activity.^1,2^ It is estimated that millions of people might be negatively affected by the neurotoxic activity of Hg,^3,4^ specifically in its main organic form, methylmercury (MeHg). Despite being present at very low concentrations in seawater, MeHg is bioaccumulated and biomagnified in aquatic food webs and may be ingested by humans mainly through the consumption of contaminated seafood.^5,6^ Despite global initiatives to stop Hg increasing levels, such as the 2013 Minamata convention,^6^ and a global state of awareness that MeHg is a neurotoxin to which large populations are being exposed, there are fundamental gaps in our mechanistic understanding of the biological processes involved in MeHg formation and degradation in the ocean.

A comprehensive inventory of Hg species in the upper 1000 m of the ocean reveals distinct concentration patterns across ocean basins.^7^ In the central and eastern tropical Pacific Ocean and the Labrador Sea, total mercury levels are below 1 pM.^7^ Conversely, the Atlantic, Arctic, and Antarctic Oceans, along with the northeast Pacific, demonstrate higher average concentrations exceeding 1 pM within the first 1000 m depth.^7^ On the other hand, MeHg concentrations in surface waters exhibit variability, ranging from 3 to 34% of total Hg. In the Arctic and eastern North Pacific, MeHg levels are comparatively lower, ranging from 3% to 13% and 4% respectively, predominantly as monomethylmercury (MMHg).^7^ In contrast, the North Atlantic consistently presents higher and fluctuating MeHg levels, averaging 34% ± 22%, with MMHg as the dominant species.^7^ The South Atlantic shows MeHg constituting 4–5% of total Hg.^7^ Despite this valuable data, a notable knowledge gap persists regarding the measurements of MeHg, particularly MMHg at depths ranging from below 1000 to 4000 m depth at a global scale, with only a few studies showing concentrations of the mentioned species.^8−13^ This deficiency is compounded by the analytical difficulties in detecting MeHg at ultra-trace levels in waters, as well as the scarcity of research cruises dedicated to the simultaneous study of trace metals and microbial communities, further impeding comprehensive understanding in the deep ocean.

The global distribution of MeHg in the oceans is influenced by three primary processes: (a) the methylation of inorganic mercury (Hg^II^), leading to the formation of MeHg; (b) the subsequent demethylation of MeHg to inorganic mercury (from MeHg to Hg^II^); and (c) the degradation of dimethylmercury.^2^ Most studies so far have focused on the understanding of the biological Hg^II^ methylation in surface oxic layers^14−21^ and pelagic redoxclines,^20,22,23^ and less attention has been paid to biological processes involved in Hg^II^ reduction and MeHg demethylation in the ocean.^24^ Both Hg^II^ reduction and MeHg demethylation processes can be abiotically (i.e., photochemically)^25−28^ or biotically mediated.^29−31^ Although photochemical Hg^II^ reduction has often been considered for near-surface waters,^27,28^ it is likely that biological Hg^II^ reduction and MeHg demethylation dominate in dark layers. Biological reduction of Hg^II^ has mostly been reported in freshwater systems,^32−35^ in the High Arctic,^36,37^ yet only a few times in the ocean.^24,38^ Consequently, very little is known about the distribution and relevance of Hg cycling microorganisms in marine waters, or the drivers controlling their presence and activity, especially in the dark ocean.

The biological Hg^II^ reduction and MeHg demethylation are carried out by a diverse group of bacterial and archaeal lineages that contain the Hg resistance system *mer* operon.^39^ The operon encodes several *mer* genes and codifies the action of a mercuric reductase (MerA, which reduces Hg^II^ to Hg^0^) and the organomercurial lyase (MerB, which demethylates MeHg into Hg^II^).^40,41^ The complete demethylation pathway of MeHg is usually achieved by the combined action of both MerA and MerB enzymes. Recently, *merA* and *merB* genes have been screened in 84 032 publicly available prokaryotic genomes (isolates, single-cell genomes, and metagenome-assembled genomes) and they have been detected in 55 bacterial and archaeal phyla.^39^ Despite having *merB* genes confer resistance to MeHg and therefore represent an ecological advantage, display a narrower phylogenetic distribution than merA.^37,41^ The latter is estimated to be ten times more abundant than *merB* in the environment.^42^ The role of *merA*, and to a lesser extent *merB*, in soils,^43^ freshwater,^44^ and highly polluted ecosystems^45,46^ are relatively well-known, and usually, the presence, and particularly, the expression of *merA* and *merB* genes have been associated with high Hg concentrations. In the ocean, as we mentioned, Hg^II^ and MeHg concentrations are generally low, and only a few studies focusing on polar sea waters and the equatorial Pacific Ocean^24,37,38^ have locally detected the genes involved in Hg detoxification in marine ecosystems. However, a recent study reported that *merA* and *merB* of *Alteromonas* and *Marinobacter* spp. bacterial isolates, capable of demethylating MeHg and reducing Hg^II^ forms, were widespread in surface, the deep chlorophyll maximum, and mesopelagic waters of different oceanographic regions,^47^ suggesting the relevance of these processes, although rarely reported. Nevertheless, the global diversity, biogeography, activity, and especially the environmental drivers of the microorganisms involved in Hg^II^ reduction and MeHg demethylation in the bathypelagic realm remain largely unknown.

The bathypelagic ocean is primarily characterized by low concentrations of labile dissolved organic carbon compounds, with a fraction of prokaryotes associated with sinking particles.^48,49^ Free-living (FL, 0.2–0.8 μm) and particle-attached (PA, 0.8–20 μm) marine prokaryotic communities are known to differ in their diversity, structure,^50,51^ functional composition,^52^ and dominant metabolisms.^52^ Given that the bathypelagic is considered an energy-limited system due to the low concentration of organic resources and that Hg detoxification is an energy-consuming mechanism,^53^ we hypothesize that *merA* and *merB* genes, widespread in the global ocean, would be more abundant in particle-attached communities. Other environmental factors may also control the global biogeography and expression patterns of *merA* and *merB* genes in the deep ocean. To test this hypothesis and provide a new understanding of the biological Hg cycle in the ocean, we analyzed 52 global metagenomes (metaG, 26 stations) and 26 metatranscriptomes (metaT, 12 stations) from the Malaspina Expedition 2010^54^ representing FL and PA prokaryotic communities, covering different oceanic regions, and spanning large environmental gradients. This study provides a global description of *merA* and *merB* genes in the tropical and subtropical bathypelagic ocean, including the Atlantic, Pacific, and Indian Oceans. In particular, we (i) describe the genetic potential of bathypelagic microbial communities for MeHg demethylation and Hg^II^ reduction (through the analyses of abundance/presence of *merA* and *merB* genes), (ii) reveal the transcription of these genes in the bathypelagic realm; (iii) elucidate the environmental factors explaining the biogeography and transcription levels of those genes; and (iv) unveil the taxonomic affiliation of the prokaryotes harboring *mer* genes. Detecting the MeHg demethylation and Hg^II^ reduction processes in the ocean proves challenging with conventional analytical methods. Hence, our study revealing the presence and transcription of *merA* and *merB* genes in the global deep ocean underscores the significance and potential global relevance of these two processes.

## Materials and Methods

### Origin of the Samples

Samples were obtained from the Malaspina Expedition 2010,^54^ comprising 26 metaG^52^ and 12 metaT datasets (this last one is a new dataset generated in this study) with free-living (0.2–0.8 μm) and particle-attached (0.8–20 μm) size fractions. In some analyses of metaT data, additional samples from 11 stations (having only one of the size fractions) were also included. All samples were collected within the bathypelagic realm (average depth: 3731 m ± 495; standard deviation). Station locations are depicted in Supplementary Figure S1, and station metadata are available in Supplementary Table S1. Details regarding specific sampling conditions, seawater filtering, library preparation, and sequencing for DNA have been previously presented.^52,55^

Sampling recovery and on-board processing of the deep ocean waters took about 3–4 h. The samples were kept close to their original temperature (∼4 °C) on board. We are aware that long sampling times and depressurization during sample recovery from the deep ocean may alter the original RNA pool from the community, but the fact that all samples were processed using the same protocol any bias will occur similarly between them, and still comparative analyses can be performed between them. RNA was extracted from 12 L of seawater sequentially filtered (within ∼15 min) using 0.8 and 0.2 μm pore size 142 mm polycarbonate membrane filters (Merk Millipore, Isopore polycarbonate, Germany). The filters were then flash-frozen in liquid N_2_ and stored at −80 °C until extraction. RNA was extracted with the RNEasy kit (Qiagen, The Netherlands) following the manufacturer’s instructions. Residual DNA was removed using a Turbo DNA-free kit (Applied Biosystems, Austin, TX, USA), and the absence of rDNA in the RNA sample was verified through PCR with universal 16S primers. RNA was reverse-transcribed using random hexamers and the SuperScriptIII kit (Invitrogen, Fisher Scientific) according to the manufacturer’s instructions. MetaT was sequenced by the DOE′s Joint Genome Institute (JGI).

### Deep-MalaspinOmics Dataset and Data Processing

The processing of metaG sequence data was previously described in Acinas et al.^52^ All predicted genes larger than 100 bp from the 52 metaG samples were clustered at 95% sequence similarity and 90% sequence coverage with cd-hit-est v4.6.6^56^ to build a nonredundant deep ocean reference catalog containing 1 115 269 nonredundant gene sequences (Deep Ocean Microbial Reference Gene Catalog; DOM-RGC)^52^. Nonredundant genes were annotated using the NCBI CDD database, COG (v1.0), PFAM database (release 28.0), and KEGG database (release 2015-10-12), and their taxonomy was estimated with Rapsearch v2.22 (Ye et al.^57^) against UniRef100 (release 2015-10).

Additionally, we included in this study a new dataset of metaT (2–8 Gb of sequencing depth). First, the reads were clipped from adapters and trimmed using Trimmomatic v3.6 (Bolger et al.^58^) with a minimum read length score threshold of 20, a sliding window of 4 bp, and a minimum read length of 50 bp. The ribosomal fraction (rRNA) and nonribosomal fraction (nrRNA) were filtered using SortmeRNA v2.1 (Kopylova et al.^59^) with its built-in Silva v119 and Rfam v11.0 databases. Detailed information on the stations studied, filter size, date, ocean, longitude, latitude, depth, salinity, temperature, oxygen, water mass, and oceanographic basin can be found in Supplementary Table S1.

### Building Hidden Markov Models for Mining *merA* and *merB* Genes

Within the *mer* operon, *merA* serves as the essential marker gene, while *merB,* which confers resistance to MeHg, is less common within the operon.^41^ MerA and MerB present challenges in functional annotation due to their high similarity with functionally unrelated paralogs and the high diversity between orthologs.^37,60^ To overcome these challenges and ensure accurate annotation, new multiple-specific hidden Markov models (HMM) were applied to detect both *merA* and *merB* genes (Supplementary Figure S2). We retrieved the *merA* and *merB* sequences from UniProtKB using the KEGG identifiers K00520 and K00221, respectively. We utilized our in-house Python tool, “merVerifyer,” which employed the *merA* sequence from *Bacillus* sp. RC607 (NCBI accession number BAB62433) or the *merB* sequence from *E. coli* (UniProt accession number P77072) as a reference. This tool verified that all *merA* and *merB* sequences contained the essential amino acids required for enzyme activity^42^ (see Supplementary Figure S2). For *merA,* the essential amino acids screened based on the *Bacillus* sp. reference sequence were two cysteines at positions 207 and 212, a vicinal cysteine pair at the carboxy terminus positions 628–629, a tyrosine at position 264, and a tyrosine at position 605 for bacteria or phenylalanine at position 605 for archaea. On the other hand, for *merB,* the essential amino acids screened based on the *E. coli* reference sequence were a tyrosine at position 93 and a cysteine at position 159. More details regarding HMM construction are explained in the Supporting Information.

### Abundance and Expression Levels of *merA* and *merB*

To determine the abundance and expression of *merA* and *merB* genes, trimmed metaG and metaT reads were mapped against the DOM-RGC with Bowtie2 (Bowtie2 v2.3.1; -X 600-rdg 6,5-rfg 6,5-score-min L,-.6,-.4-no-discordant no-mixed).^61^ Samtools (v1.3)^56^ was used to sort the alignments by read name and compress the alignment files. Metagenomes and metatranscriptomes read alignments were filtered based on the *merA* and *merB* annotation obtained with HMM. Effective counts of *merA* and *merB* genes in the metaG and metaT were normalized by essential single-copy genes (SCGs), calculated as the arithmetic mean of effective counts of the RecA protein (*recA*), DNA-directed RNA polymerase subunit beta (*rpoB*), and DNA gyrase subunit B (*gyrB*). This normalization approach accounts for variations in microbial biomass and activity among samples, facilitating a more accurate comparison of the expression levels of *mer* genes by aligning them with the bacterial cellular unit. The taxonomic affiliation of *merA* and *merB* sequences was curated using the previously published Christakis et al.^39^ database and phylogeny (see the Supporting Information for a detailed explanation).

### Environmental Variables and Characterization of the Dissolved Organic Matter

The following variables were tested to determine which environmental drivers could shape the *mer*-coding prokaryotic communities: temperature, salinity, apparent oxygen utilization (AOU), concentrations of inorganic nutrients (NO3-, PO4-), and the fluorescent dissolved organic matter (DOM) components C1, C2, C3, and C4 (Supplementary Table S1). Dissolved organic matter was characterized through the optical properties of its fluorescent fraction, which provides information about the origin and lability of DOM. These optical properties were described based on fluorescence excitation/emission matrices (EEMs) as explained in previous studies.^62−64^ Four main fluorescence components were recovered from the EEMs using parallel factor analysis (PARAFAC): Components C1 and C2, previously related to refractory humic-like material, and C3 and C4, which represent more biodegradable and fresher microbially produced FDOM (for more details, see previous studies^62−64^).

### Statistical Analyses

To determine significant differences between *merA* and *merB* gene abundance and expression across ocean basins, as well as between lifestyles, the Shapiro-Wilk test from the *stats* package of the R software^65^ was used to assess the normality of the data distribution. Since our dataset did not fit into a normal distribution, we employed the Kruskal–Wallis test to assess statistical significance between groups, and posthoc multiple comparison analyses (if Kruskal–Wallis turned significant) by pairwise Wilcox tests. To visualize similarities in *merA* and *merB* containing communities, nonmetric multidimensional scaling analyses (nMDS) were performed using the *vegan* package.^66^ Additionally, the influence of environmental variables shaping the abundance and expression of *merAB* genes was explored using Mantel tests correlations between a Bray–Curtis taxonomic dissimilarity matrix and a matrix with Euclidean distances of the different environmental parameters. PERMANOVA test was applied between the Bray–Curtis taxonomic dissimilarity matrix and Euclidean distances to detect if changes in the environmental variables determine significant changes in *mer*-containing communities (*p*-value < 0.05).

### Data Availability

Malaspina Bathypelagic metagenomes can be accessed in the ENA repository under accession number PRJEB44456, while metatranscriptomes under accession number PRJEB76155. The custom HMM and *merA* and *merB* gene FASTA sequences detected have been deposited in BioStudies under accession number S-BSST1154. Our in-house Python tool “merVerifyer” has been deposited in the GitLab repository (https://gitlab.com/pablo.sanchez/merverifyer).

## Results and Discussion

### Abundant and Active *merA* and *merB* Genes Across Deep Ocean Basins

**Figure 1:**
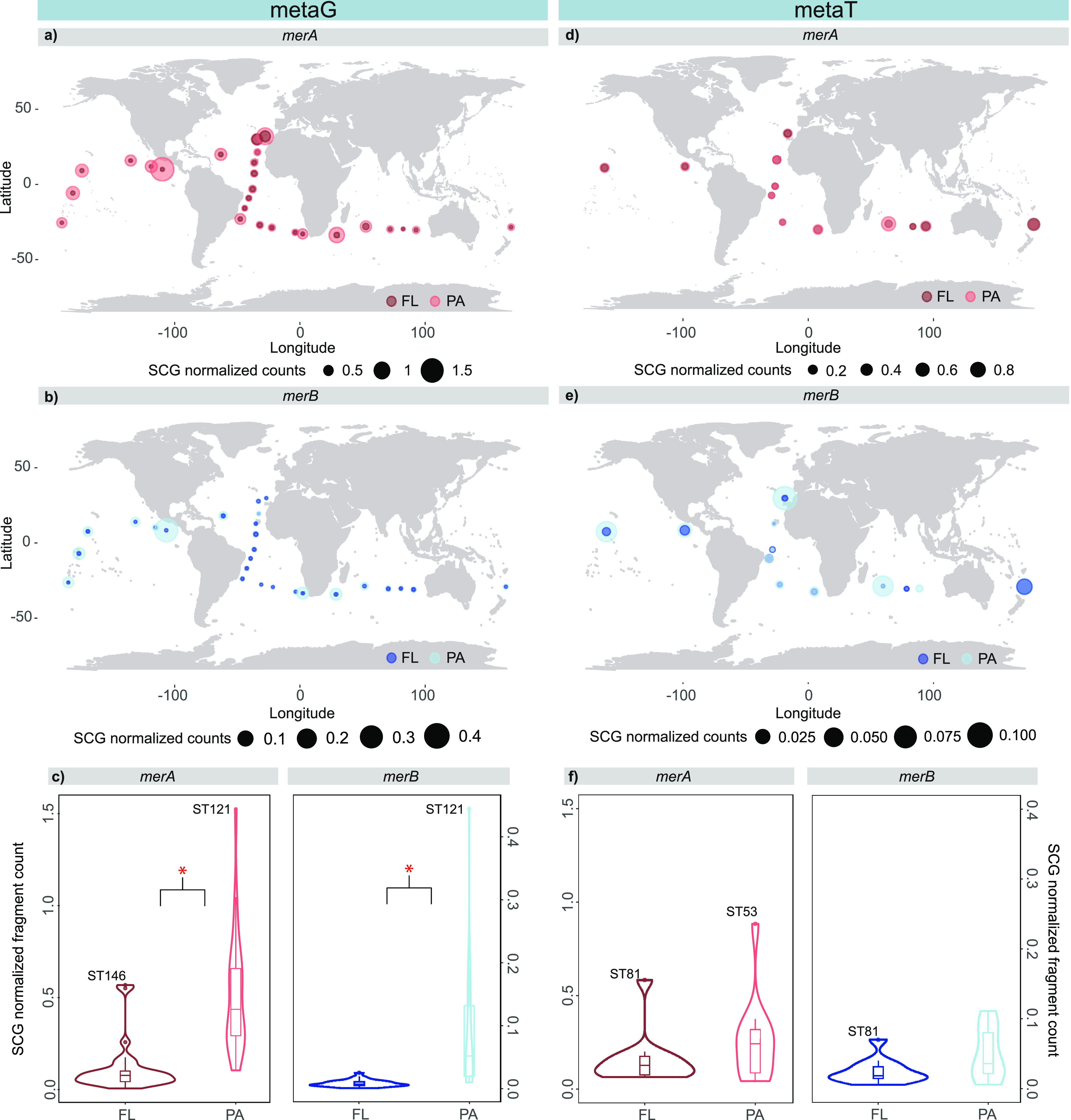
Mercury detoxification genes are widespread in the deep global ocean. Relative abundance of metagenome-derived (metaG, *n* = 26 stations, 52 samples) *merA* and *merB* genes (a–c) and metatranscriptome-derived (metaT, *n* = 12 stations, 26 samples) *merA and merB* genes (d–f) in each size-fraction (FL: free-living; PA: particle-attached) and across the studied bathypelagic stations. The size of the dots represent abundances in metaG or metaT normalized by counts of single copy genes (SCG). The color of the dots indicates size fraction: dark blue and dark orange for FL; light blue and light orange for PA. Red asterisks indicate significant differences between size fractions (Kruskal–Wallis, *p*-value < 0.001). Outlier stations with the highest abundances are labeled in the boxplots. Note that the scales in the *merA* and *merB* boxplots differ in the metaG and metaT data.

Exploring 52 metaG samples from 26 stations of the Malaspina Expedition (Supplementary Figure S1), we determined the presence and the abundance of *merA* and *merB* genes in the free-living (FL 0.2–0.8 μm; *n* = 26) and particle-attached (PA: 0.8–20 μm, *n* = 26) prokaryotic communities. In 12 of these 26 stations, we also evaluated the expression of these genes based on the metaT data from both FL and PA size fractions. Our results, hereafter expressed as per-cell average abundance, normalized by single-copy genes, showed that *merA* and *merB* genes were ubiquitous (metaG) (Figure 1a–c) and expressed (metaT) (Figure 1d–f) in the bathypelagic realm of all studied ocean basins, including the Pacific, Atlantic and Indian Ocean. However, we observed contrasting differences between *merA* and *merB* gene abundance, with comparatively higher abundances of the *merA* gene (Kruskal–Wallis, *p*-value < 0.001) (Figure 1c). The larger abundance of *merA* than *merB* genes in the bathypelagic realm aligns with the observation that *merA* is ten times more abundant than *merB* in genomes derived from cultured isolates, metagenome-assembled genomes, and single-cell genomes.^39,42^ Although recent findings identified *mer* operon with only *merB* gene^39^ the higher abundance of *merA* genes seems coherent since inorganic Hg concentrations are generally higher than MeHg in the ocean.^7^ Interestingly, metaT data revealed no significant differences in expression between *merA* and *merB* genes (Kruskal–Wallis, *p*-value > 0.05) in both size fractions (Figure 1f), indicating that both genes were actively transcribed at similar rates across ocean basins and that most bacteria encoding for the *merA* genes did not express it.

Notably, peaks of abundance of *merA* and *merB* genes were detected in ST121 from the North Pacific Ocean in the PA size fraction and the FL fraction for ST146 (only for *the merA* gene). Noteworthy, high expression levels of *merA* were found in the FL fraction of ST81 from the South Pacific Ocean and in the PA fraction of ST53 from the Indian Ocean. Hence, increased gene abundance does not correlate with an increase in gene expression (Supplementary Figure S3), a trait previously reported for some ecological functions in microbial communities, such as genes involved in sulfate reduction processes,^67^ or for the Hg^II^ methylating genes (*hgcAB*) in Baltic sediments.^68^ Our results not only report the prevalence but also its expression in the tropical and subtropical global deep ocean in both plankton size fractions (FL and PA) and suggest that both demethylation and reductive volatilization might occur even at low Hg^II^ and MeHg concentrations.

### Different *merA* and *merB* Coding Prokaryotic Communities Dominate the Free-living and Particle-attached Fractions in the Deep Ocean Across Different Basins

**Figure 2:**
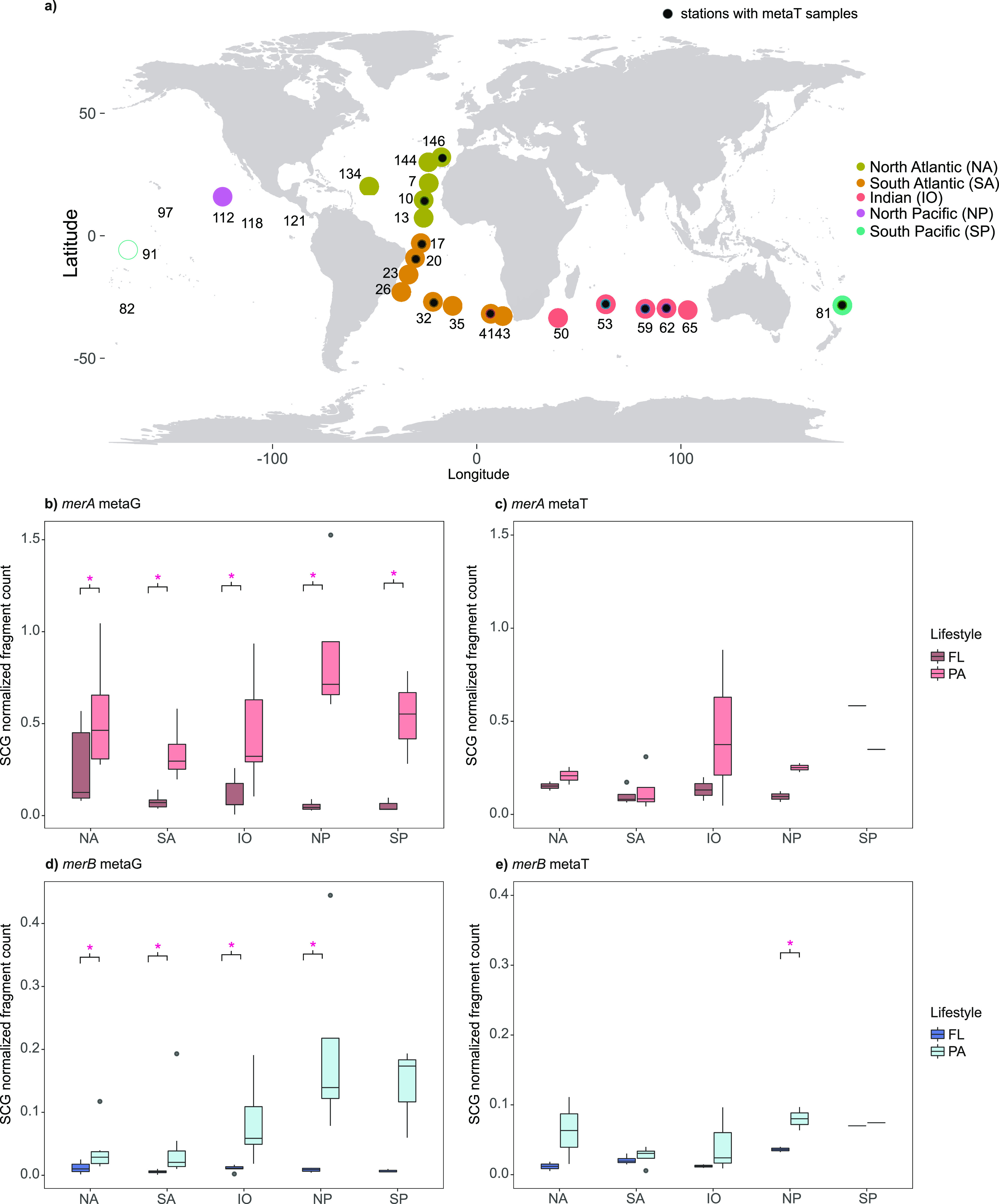
Relative abundance of the *merA* and *merB* genes retrieved in each ocean basin. (a) Map indicating the position of the metagenomes (metaG), colored by ocean basin, and metatranscriptomes (metaT), indicated by black dots. (b,d) Relative abundances normalized by single-copy genes (SCG) in the metaG, and (c,e) in the metaT. Only one station was kept for analyses in the South Pacific metaT data, represented by a single line in the boxplots. FL: free-living, PA: particle-attached. Dark blue and dark orange: FL; light blue and light orange: PA. Red asterisks show significant differences between size fractions (Kruskal–Wallis, *p*-values < 0.05).

In this study, we observed a significantly higher gene abundance of *merA* (Kruskal–Wallis, *p*-value < 0.001) and *merB* (Kruskal–Wallis, *p*-value < 0.001) in the PA fraction compared to the FL across ocean basins (Figure 2). Gene *merA* consistently showed higher abundance in the particle-attached (PA) fraction, as depicted in Figure 2b and detailed in Supplementary Tables S2 and S3. On the other hand, while *merB* also exhibited higher abundances in the PA fraction compared to the free-living (FL) fraction, the disparities were particularly pronounced in the Indian and Pacific Oceans. When examining the expression level, we observed slightly higher expression in the PA fraction compared to the FL, but globally no significant differences were detected for the two genes between the two lifestyles. An exception to this was the transcript levels of *merB* genes in the North Pacific, where higher expression was detected in the PA fraction (Kruskal–Wallis, *p*-value < 0.001) (Figure 2c,e and Supplementary Table S2). Nonmetric multidimensional scaling analyses (nMDS) based on metaG abundances of *merA* and *merB* genes also showed a clear division between FL and PA prokaryotic communities (PERMANOVA, *p*-value < 0.05) and indicated differences in terms of *merA* and *merB* community composition between oceanic regions (PERMANOVA, *p*-value < 0.01) (Supplementary Figure S4 and Supplementary Table S4). Shifts in *mer*-coding community composition between PA and FL lifestyles are consistent with previous studies that showed clear taxonomic segregation in terms of abundance and also activity of FL and PA prokaryotes in deep ocean waters, with some groups showing preferences for either one or the other lifestyle.^50,51,69^ Regarding expression levels based on metaT data collected in 12 of the 26 studied stations, we also detected differences between oceans (PERMANOVA, *p*-value < 0.05) (Supplementary Figure S4 and Supplementary Table S4), but we did not observe such clear differentiations between lifestyles. Differences in metaT expression values between size fractions were significant for *merA* (PERMANOVA, *p*-value < 0.05) but not for *merB*. Our results showed that a higher abundance of *merA* and *merB* genes in the PA communities was not systematically related to a higher expression in the same fraction (Supplementary Figure S3), suggesting that local factors, rather than the lifestyle, controlled the expression of *merA* and *merB* genes and thus, likely, the rates of Hg^II^ reduction and MeHg degradation.

### Water Mass Age and Composition of Organic Matter Shape the Abundance and Expression of *merA* and *merB* Genes in Deep Ocean Basins

Microbially mediated MeHg formation is influenced by a wide variety of environmental factors such as temperature, pH, redox potential, biological mechanisms, presence and quality of inorganic and organic matter, and biological mechanisms, which govern microbial activity and Hg^II^ bioavailability.^70−73^ However, little is known about the environmental factors controlling the microbial MeHg demethylation^74^ and especially those affecting the abundance and expression of *mer*-coding prokaryotic communities in marine ecosystems. We explored variations in different physicochemical and biological parameters such as water mass type, ocean basin, temperature, salinity, inorganic nutrients, apparent oxygen utilization (AOU), or the composition of dissolved organic matter (DOM) (Supplementary Table S1) to elucidate which environmental factors could explain the diversity and expression patterns of *mer*-coding prokaryotic communities. The composition of DOM was assessed by examining the optical properties of the fluorescent fraction of DOM (FDOM), which provides insights into the nature of the fluorescent refractory DOM pool (see Material and Methods section and previous studies^62−64^ for more details).

**Figure 3:**
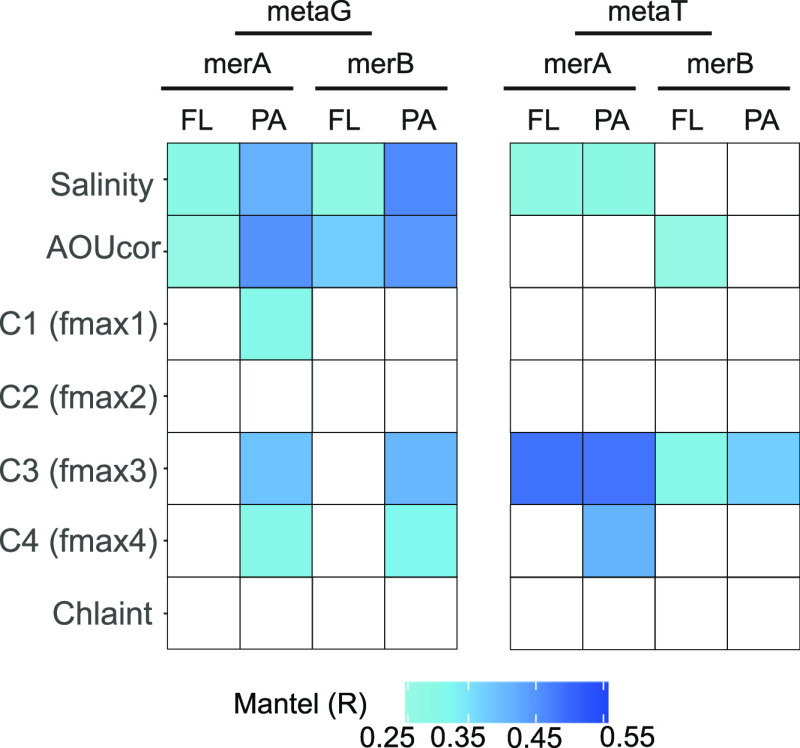
Mantel test correlating the environmental factors with the metaG or metaT data (Bray–Curtis distances between samples). Only significant Mantel R values (*p*-value < 0.01) are indicated. Darker values show higher correlation values. AOUcor: apparent oxygen utilization. C1, C2, C3, and C4: four main fluorescence DOM components (FDOM), where components C1 and C2 are related to refractory humic-like material, and C3 and C4 represent more biodegradable and fresher microbially produced FDOM (for more details see previous studies^62−64^). Chlaint: integrated chlorophyll values. FL: free-living; PA: particle-attached.

**Figure 4:**
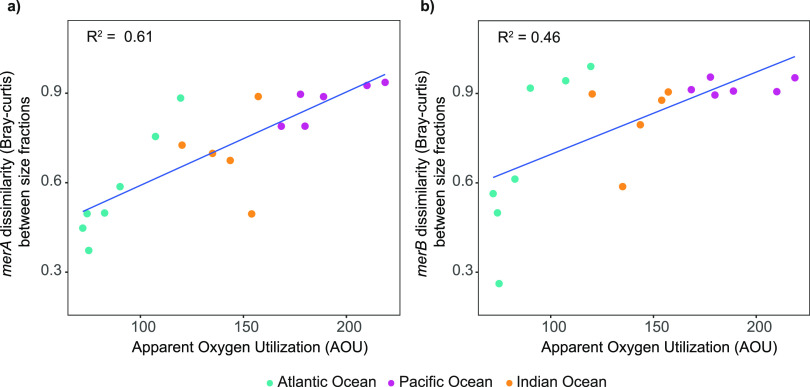
Metagenomic Bray–Curtis dissimilarity values between size fractions of the same station versus apparent oxygen utilization (AOU). Values extracted from metaG data. (a) *merA* gene. (b) *merB* gene. Colors indicate ocean basin of the station. *R*^2^ is indicated in each plot.

The differences in diversity and gene abundance between samples, both in FL and PA communities, were correlated with salinity and apparent oxygen utilization (AOU), indicative of the physicochemical properties of different water masses (as determined by Mantel tests, Figure 3). Certainly, variations in the AOU had a significant relationship (PERMANOVA, *p*-value < 0.05, Figure 4 and Supplementary Table S4) to the differences observed between stations for both genes. AOU represents the difference between the saturation and measured dissolved oxygen and is commonly used as a proxy for the water mass age^64,75^ because it integrates all respiratory processes since the water mass was last in contact with the atmosphere (surface). In our study, we observed that the physicochemical properties of the different water masses, especially apparent oxygen utilization (AOU), influenced the *mer*-coding prokaryotic communities (Figure 3). We also observed that FL and PA *merA* and *merB* harboring communities in each station were taxonomically more similar to each other in younger waters (i.e., lower AOU values), such as in the Atlantic Ocean, than in older Pacific Ocean waters, where the Bray–Curtis dissimilarity between FL and PA communities was higher (Figure 4). Indeed, it is not surprising as it has been recently reported that water aging (AOU) is one of the main environmental drivers shaping the taxonomic composition and transcription differences between prokaryotic communities in the deep ocean.^55,76^

**Figure 5:**
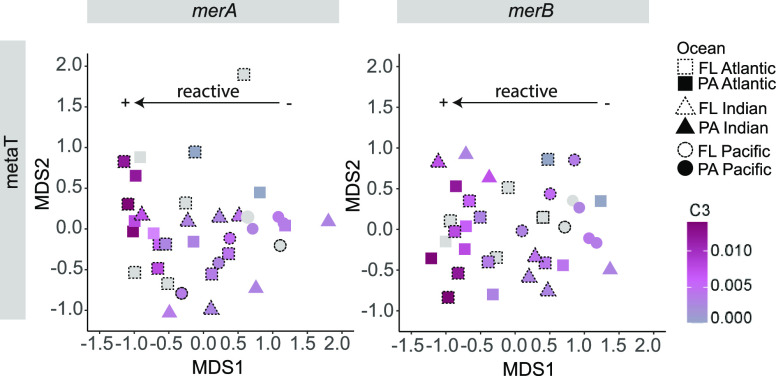
Dissolved organic matter shapes the expression of prokaryotic communities coding for *merA* and *merB* genes. nMDS plots colored by labile C3 protein-like DOM determined in FDOM analyses in metatranscriptomes (metaT) data. Symbols with dashed lines indicate free-living (FL) while solid lines indicate particle-attached (PA) size fraction samples. Higher intensity of the color indicates a higher presence of C3 protein-like DOM. Gray symbols indicate stations where environmental data was not available.

On the other hand, changes in FDOM, especially those of protein-like DOM (fluorescence of amino acid-like components C3 and C4) identified as labile in previous studies,^62,63,77^ and more precisely changes in the FDOM C3 component likely explained the expression of *mer* genes (metaT) (Figure 5, PERMANOVA, *p*-value < 0.05). We also observed that communities from stations showing higher amounts of labile C3 protein-like DOM clustered together regardless of the ocean and/or lifestyle (Figure 5). The results revealed a significant relationship between the expression of *mer* genes in the *mer*-containing community and the concentration of C3 protein-like DOM (PERMANOVA, *p*-value < 0.05, Supplementary Table S4). Our findings support previous evidence that the composition of the DOM plays an important role in driving the activity of the bathypelagic microbiome^55,77^ in different ocean regions and lifestyles. The composition of organic matter was previously identified as a key driver of MeHg formation rates^71^ and of the communities involved in MeHg formation^78^ in lakes and also in coastal waters.^73,79^ In open waters, MeHg formation has been associated with the regeneration of organic matter in the Mediterranean^21^ and Baltic seas.^80^ This study highlights the relevance of organic matter composition for MeHg degradation and thus provides new linkages between DOM and Hg cycling in the ocean.

Our results indicate that different environmental factors control the diversity and the expression of *mer*-coding prokaryotic communities in the deep ocean. It has to be noted, though, that metaT analyses provide a snapshot of gene expression under specific conditions at the time of sampling, implying that the activity is more variable than the distribution of *mer*-coding bacteria. This variability would explain why different environmental parameters are shaping the abundance or the expression of *mer* genes. Studies focusing on the environmental factors affecting the biotic demethylation processes mediated by *mer*-coding communities are scarce, and we have been able to report which factors may influence *mer*-coding microbial communities and the expression of *merA* and *merB* genes in the ocean.

### Main Taxonomic Players Involved in Hg Detoxification in the Deep Ocean

#### Alteromonadales and Corynebacteriales Containing *merA* Gene Dominate in the FL and PA Fractions Respectively

**Figure 6:**
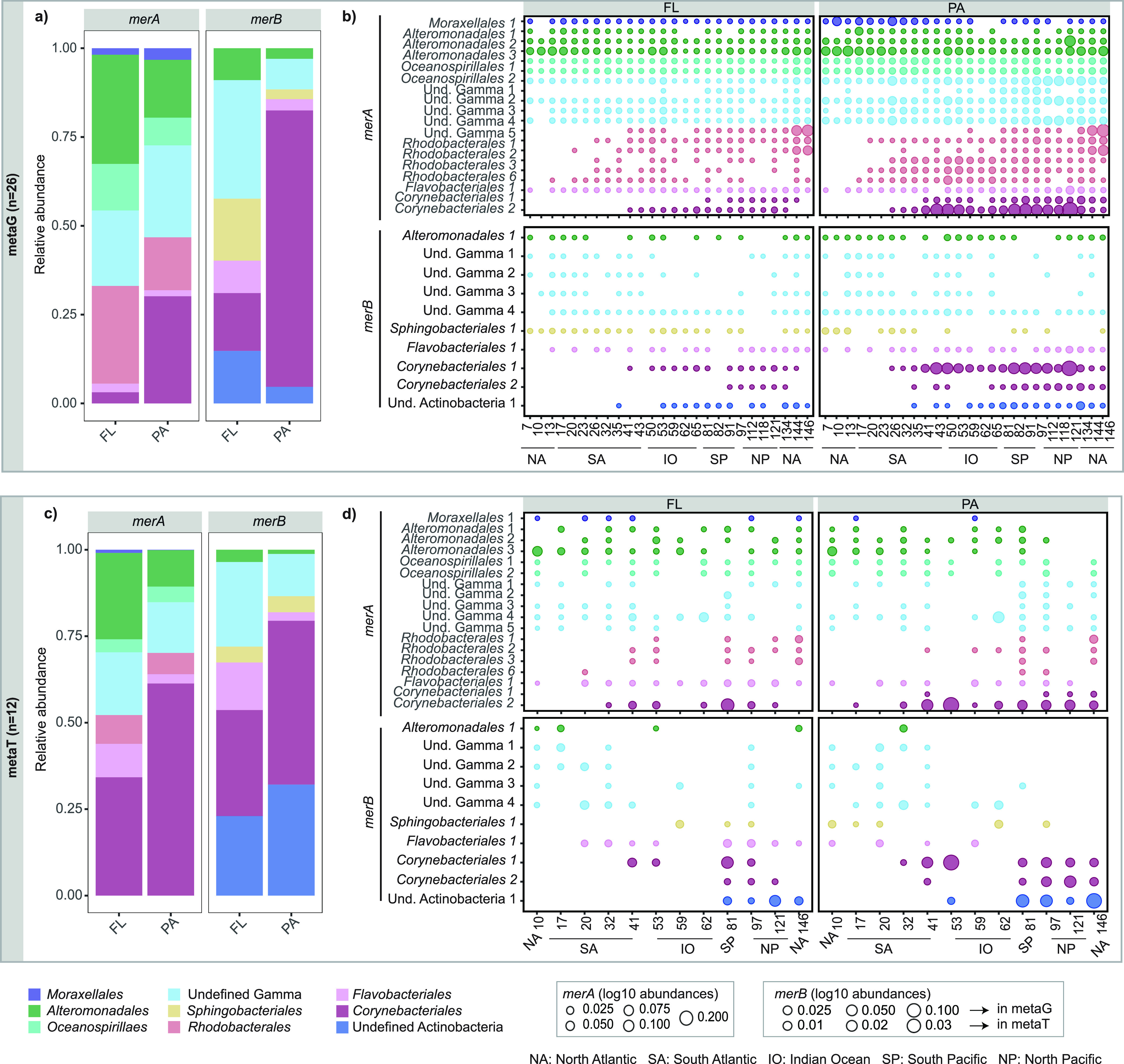
Taxonomic affiliation of the *merA* and *merB* gene variants. (a,c) Relative abundance in the metaG (diversity and abundance) and the metaT (expression) for FL and PA bacterial communities. Colors indicate different taxonomic groups at the order level. (b,d) Abundance of the different taxonomically identified *merA* and *merB* gene variants in the FL and PA communities in the metaG and metaT datasets. Colors indicate different taxonomic groups at the order level. The size of the dots indicate log10 scale of the abundances normalized by single-copy genes (SCG) of *merA* and *merB* genes. Note that for *merA* legend size is the same for metaG than for metaT but it is different for *merB*. Samples on the *x*-axis are ordered by ocean basin: NA, North Atlantic Ocean; SA, South Atlantic Ocean; IO, Indian Ocean; NP: North Pacific Ocean; SP, South Pacific Ocean.

In total, 20 *merA* sequence variants were detected, most of which affiliated with the Alphaproteobacteria class, specifically to the *Rhodobacterales* order with six sequence variants (30%), followed by Gammaproteobacteria class, including three *Alteromonadales* sequences (15%), two *Oceanospirillales* (10%), one *Moraxellales* (5%), and five that could be not further classified (25%) at the order level. Additionally, we found two *merA* sequences belonging to the Actinobacteria phylum and one to the Bacteroidetes phylum, from *Corynebacteriales* (10%) and *Flavobacteriales* orders (5%), respectively (Figure 6a,c, Supplementary Table S5 and Supplementary Figure S5). These taxonomic groups had been previously reported as *merA*-coding bacteria in the environment^39^ such as in soils,^81^ the Arctic and Pacific Oceans,^24^ and in cultured isolates from snow, sea-ice, and freshwater,^37^ sediments,^82^ soils,^83^ or hydrothermal vents.^84,85^ Curiously, we could not detect any archaeal *merA* sequence. Different reasons could account for this phenomenon. First, archaeal microorganisms present in the deep ocean (usually accounting for up to 16% of the total prokaryotic community^50,52^), are primarily found in the FL fraction, rather than in the PA fraction, which represents half of our dataset. Second, due to the moderate sequencing effort in these samples (3.36 Gb/sample,^52^), it is possible that we may have overlooked them. Additionally deep ocean archaea may not harbor *mer* genes or are present at very low abundances. It is known that archaea containing these genes are typically found in specific environments such as hydrothermal vents or acidic hot springs.

In terms of abundance, all taxonomic orders detected among the *merA* gene variants presented significant differences between FL and PA size fractions (Wilcoxon test, *p*-value < 0.001) with *Alteromonadales* presenting the highest abundances in the FL, and *Corynebacteriales* in the PA fraction (Figure 6a, Supplementary Table S6 and Figure S6). Nevertheless, no significant differences were generally observed between *merA* sequence variants at the expression level between size fractions (Figure 6c), except for only *Oceanospirillales* despite their low expression numbers (Wilcoxon test, *p*-value < 0.01) (Supplementary Table S6 and Figure S6). Interestingly, all the *merA* sequences present in the FL fraction were also detected in the PA fraction (Figure 6b,d and Supplementary Table S7), which concurs with previous results regarding the presence of dual lifestyles for certain prokaryotes, such as *Alteromonadales* or *Oceanospirillales*, meaning that they could live both as part of the FL or PA bacterial communities.^51,55^

It is noteworthy to mention that *merA* sequence variants from the Gammaproteobacteria class (*Moraxellales*, *Alteromonadales*, and *Oceanospirillales*), as well as those classified as *Flavobacteriales* in the Bacteroidetes phylum, were generally present and widely expressed at the different sites (Figure 6b,d). However, the *merA* sequence variants from the Alphaproteobacteria class (*Rhodobacterales*) and Actinobacteria phylum (*Corynebacteriales*) exhibited a patchy distribution across different ocean regions and were absent from some Atlantic and Indian Ocean stations. This agrees with the overall distribution of these groups in the bathypelagic, as *Corynebacteriales* dominated PA communities in the Pacific Ocean but was mostly absent in other ocean basins.^51^ Furthermore, it is noticeable that some of these sequence variants were present in certain stations where they showed no detectable expression. For instance, *merA Corynebacteriales* 1 (Figure 6b,d) was detected in the metagenomes of the FL fraction of stations from the South and North Pacific Ocean, but no expression was observed in this size fraction at any of the stations. These results could be explained by the idea that bacteria show temporal variations in their expression patterns^86^ or the fact that some bacteria could persist in the bathypelagic as inactive or dormant bacteria comprising a microbial seed bank able to quickly respond to changes in the environmental conditions.^87,88^

#### Gammaproteobacteria Dominated *merB* Containing Communities of the Deep Ocean

Due to the optionality of *merB* within the *mer* operon, which is linked to the evolution of the operon by serial recruitment of functions through an evolutionary time-scale,^42^ it was expected to find fewer *merB* than *merA* genes in our bathypelagic microbial metaG and metaT datasets. Indeed, we found 14 *merB* sequence variants. They mainly associated with the Gammaproteobacteria class, with two *Alteromonadales* sequence variants (14.3%), one from the *Cellvibrionales* order (7.14%) and four that could not be classified at the order level (40%), followed by the Actinobacteria phylum with two *Corynebacteriales* sequence variants (14.3%), and two undefined Actinobacteria (14.3%). Lastly, two Bacteroidetes phylum sequence variants belonging to *Sphingobacteriales* (10%) and *Flavobacteriales* (10%) orders, and one Firmicutes sequence variant classified within the *Clostridiales* order (7.14%) (Figure 6, Supplementary Table S5 and Supplementary Figure S7). Compared to the *merA* sequence variants detected, we found a higher number of *merB* sequences that could not be classified within the Actinobacteria phylum. Interestingly, Alphaproteobacteria were not present within the *merB* sequences detected. *Sphingobacteriales* (Bacteroidetes) *and Clostridiales* (Firmicutes) were only detected in the *merB* sequence variants suggesting that some prokaryotes could potentially present only the *merB* gene as recently reported.^39^ Like for *merA* genes, all the *merB*-containing taxonomic groups detected in our study were previously described as *merB*-coding bacteria,^39,42,89^ and *Alteromonadales* include members that have been experimentally proven in the laboratory to be able to degrade MeHg at reasonably high concentrations.^47^ Once again, the absence of archaeal sequences was observed among the *merB* genes, and similar factors explained for the *merA* gene detection may account for the absence of *merB* sequences.

Only 10 out of the 14 sequence variants could be kept after normalization of the data for further analyses in order to be able to compare between samples. Actinobaceria, *Alteromonadales*, undefined Gammaproteobacteria, and *Corynebacteriales* (metaG) were significantly more abundant in the PA fraction compared to the FL (Wilcoxon test, *p*-value < 0.01, Supplementary Table S6 and Figure S6). The transcription of these *merB* genes followed the same patterns, being higher in the PA compared to the FL fraction, although the Wilcoxon test did not show significant differences, (Supplementary Table S6 and Figure S6), suggesting either that these taxonomic groups were similarly active regardless of their lifestyle, or that due to the low number of samples that could be included in the metaT analyses, it was not possible to observe differences. Our results indicate that Actinobacteria carrying *merB* sequences could have an important role in degrading MeHg in particulate organic matter, consistent with reports from other environments, such as in sediments,^90^ in which Actinobacteria members have been described to be involved in MeHg degradation. The expression of *merB* genes was in general patchier than their abundance between stations regardless of the taxonomic group, also likely explained by the lower number of stations analyzed for metaT than for metaG. Overall, our results indicate that despite *merA* and *merB*-bearing bacteria generally preferring the PA fraction, MeHg degradation and Hg^II^ reduction can be mediated by microorganisms present in both the FL and PA fractions.

### Outlook

Even though the presence, and particularly the expression, of *merA* and *merB* genes, have been generally associated with high Hg concentrations, our study reveals the widespread genetic potential to degrade MeHg and reduce Hg^II^ in deep ocean waters, where Hg^II^ and MeHg concentrations are at ultra-trace levels. Our study indicates that deep-ocean particles are rich in *merA* and *merB* bacterial communities and that a combination of environmental drivers, including the AOU of water masses and DOM composition, seems to govern the differences in abundance and expression patterns across ocean basins and lifestyles. Moreover, our work identifies important taxonomic players involved in MeHg demethylation (*Alteromonadales*, *Corynebacteriales*, *Flavobacteriales*, *Sphingobacteriales*, and Undefined Actinobacteria or Gammaproteobacteria) and Hg^II^ reduction (*Alteromonadales*, *Corynebacteriales*, *Flavobacteriales*, *Moraxellales*, *Oceanospirillales*, *Rhodobacterales* and Undefined Gammaproteobacteria), which can be found both as free-living and particle-attached communities, although their abundances and expression vary across ocean basins. Our study significantly advances current knowledge on the global Hg biogeochemical cycle by delivering a tool searching for *merA* and *merB* genes in metaG and metaT and providing evidence of biological MeHg degradation and Hg^II^ reduction, one of the largest missing pieces to understanding MeHg cycling in the global ocean.

## Supporting Information Available

Detailed description of constructed Hidden Markov Models (HMM) and phylogenetic trees analysis including their corresponding figures; supporting figures detailing stations sampled for metaG and/or metaT, and correlations between merA and merB genes in different size fractions between metaG and metaT data and extra NMDS analyses (PDF)
Supplementary metadata tables and results to give support to the statistical analyses performed in the study (XLSX)
