# Supplementary material for: Microorganisms Involved in Methylmercury Demethylation and Mercury Reduction are Widely Distributed and Active in the Bathypelagic Deep Ocean Waters
Source: Environ Sci Technol. 2024 Jul 24;58(31):13795–807. doi: 10.1021/acs.est.4c00663 (PMC11308531; doi:10.1021/acs.est.4c00663)
Supplement: Supplementary file 1 — es4c00663_si_001.pdf [file es4c00663_si_001.pdf]

Supplementary Information for

Microorganisms involved in methylmercury demethylation and mercury reduction are widely distributed and active in the bathypelagic deep ocean waters

Isabel Sanz-Sáez[1,2,§,*], Andrea G. Bravo[1,§], Joan-Martí Carreras[1], Olga Sánchez[2], Marta Sebastian[1], Clara Ruiz González[1], Eric Capo[1], Josep M. Gasol[1], Carlos M. Duarte[3], Pablo Sánchez[1], Silvia G. Acinas[1,*]

[§]These authors have equally contributed to this work

* Corresponding authors: Silvia G. Acinas (sacinas@icm.csic.es), Isabel Sanz-Sáez (isanz@icm.csic.es), and Andrea G. Bravo (andrea.bravo@icm.csic.es).

This PDF file includes:

Supplementary Material

Supplementary Figures S1 to S7

Headings Supplementary Tables S1 to S7

Other Supplementary Material for this manuscript include the following:

Excel file with Supplementary Tables S1 to S7.

Supplementary Material

Construction of Hidden Markov Models (HMM) for merA and merB genes

We retrieved MerA sequences from UniProtKB using the KEGG identifier K00520, yielding a total of 492 proteins. We eliminated redundancy by clustering these proteins with cd-hit [1], resulting in a set of 404 unique proteins. Subsequently, we utilized our in-house Python tool, "merVerifier," which employed the MerA sequence from Bacillus sp. RC607 (NCBI accession number BAB62433) as a reference. This tool verified that all *merA* sequences contained the six essential amino acids required for enzyme activity [2]. With the 377 proteins left, we rebuilt the alignment with MAFFT (default parameters), and used *hmmbuild* to create two HMM models: one with the default parameters and the other with *–pnone*, so the probability parameters of this model were not "contaminated" with priors. We manually adjusted the probabilities of essential amino acids in the first model, aligning them with those from the model without priors. This created a stricter model that focused solely on these positions.
A similar approach was used for MerB. We retrieved 60 MerB proteins from UniProtKB using its KEGG identifier K00221. After clustering, we were left with 55 proteins, and our "merVerifier" program was used to confirm that they all contained the two essential amino acids [3] with the MerB sequence from *E. Coli* (UniProt accession number P77072) as reference, discarding four proteins. We aligned the 51 proteins left using MAFFT (default parameters) and then built the HMM model with *hmmbuild* (default parameters).

Phylogenetic trees

For each phylogenetic analysis, the amino acid sequences from *mer* genes used to build the Hidden Markov Models (HMM), *mer* genes detected in obtained metagenomes and Christakis et al. (2021) [5] published *merA* and *merB* sequences were aligned using

MUSCLE. RAxML (v8.2.10) was used to generate maximum likelihood tree under the GAMMA distribution with the LG model with the following parameters: raxmlHPC -f a -p 283976 -m PROTGAMMAAUTO -N autoMRE -x 2381 -T 10 [4]. Branch support was generated by rapid bootstrapping.

REFERENCES

(1) Fu, L.; Niu, B.; Zhu, Z.; Wu, S.; Li, W. Sequence Analysis CD-HIT: Accelerated for Clustering the next-Generation Sequencing Data. **2012**, *28* (23), 3150–3152. https://doi.org/10.1093/bioinformatics/bts565.

(2) Boyd, E. S.; Barkay, T. The Mercury Resistance Operon: From an Origin in a Geothermal Environment to an Efficient Detoxification Machine. *Front Microbiol* **2012**, *3* (October), 349. https://doi.org/10.3389/fmicb.2012.00349.

(3) Pitts, K. E.; Summers, A. O. The Roles of Thiols in the Bacterial Organomercurial Lyase (MerB). *Biochemistry* **2002**, *41* (32), 10287–10296. https://doi.org/10.1021/bi0259148.

(4) Kozlov, A. M.; Darriba, D.; Flouri, T.; Morel, B.; Stamatakis, A. RAxML-NG: A Fast, Scalable and User-Friendly Tool for Maximum Likelihood Phylogenetic Inference. *Bioinformatics* **2019**, *35* (21), 4453–4455. https://doi.org/10.1093/bioinformatics/btz305.

(5) Christakis, C. A.; Barkay, T.; Boyd, E. S. Expanded Diversity and Phylogeny of Mer Genes Broadens Mercury Resistance Paradigms and Reveals an Origin for MerA among Thermophilic Archaea. *Front Microbiol* **2021**, *12*, 682605. https://doi.org/10.3389/FMICB.2021.682605/FULL.

Supplementary Figures

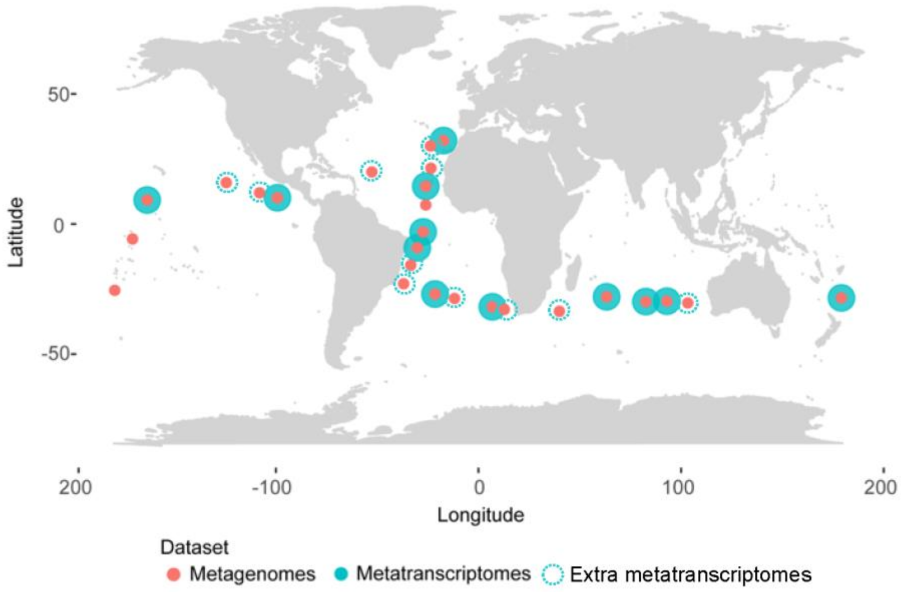

Fig. S1. World map showing the distribution of bathypelagic metagenomes (orange, n=26) and metatranscriptomes (solid blue, n=12) samples of the free-living (FL) and particle-attached (PA) size fractions used for determining the abundance and expression levels of *merA* and *merB* genes. An extra dataset of metatranscriptomes samples (dashed blue circles, n=11) for which we only had the FL or PA size fraction were included in some analyses to determine the environmental factors driving the biogeography and expression patterns of *merA* and *merB* genes.

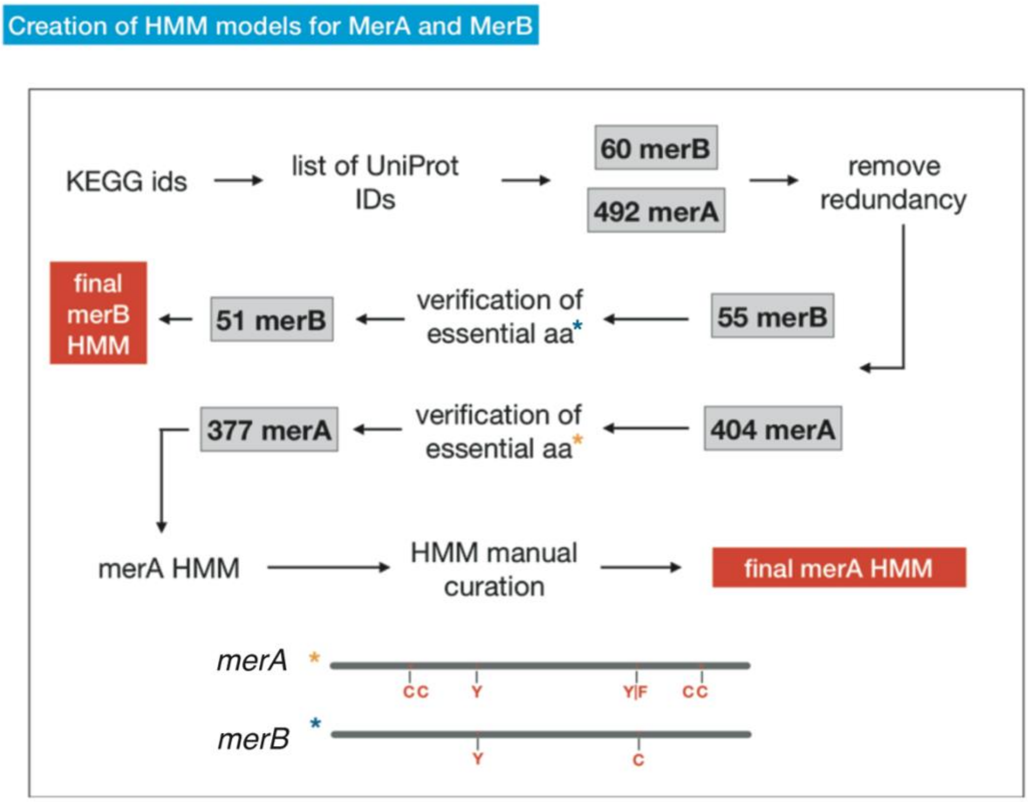

Fig. S2. Detection of *merA* and *merB* genes using Hidden Markov Models (HMM) in Malaspina datasets.

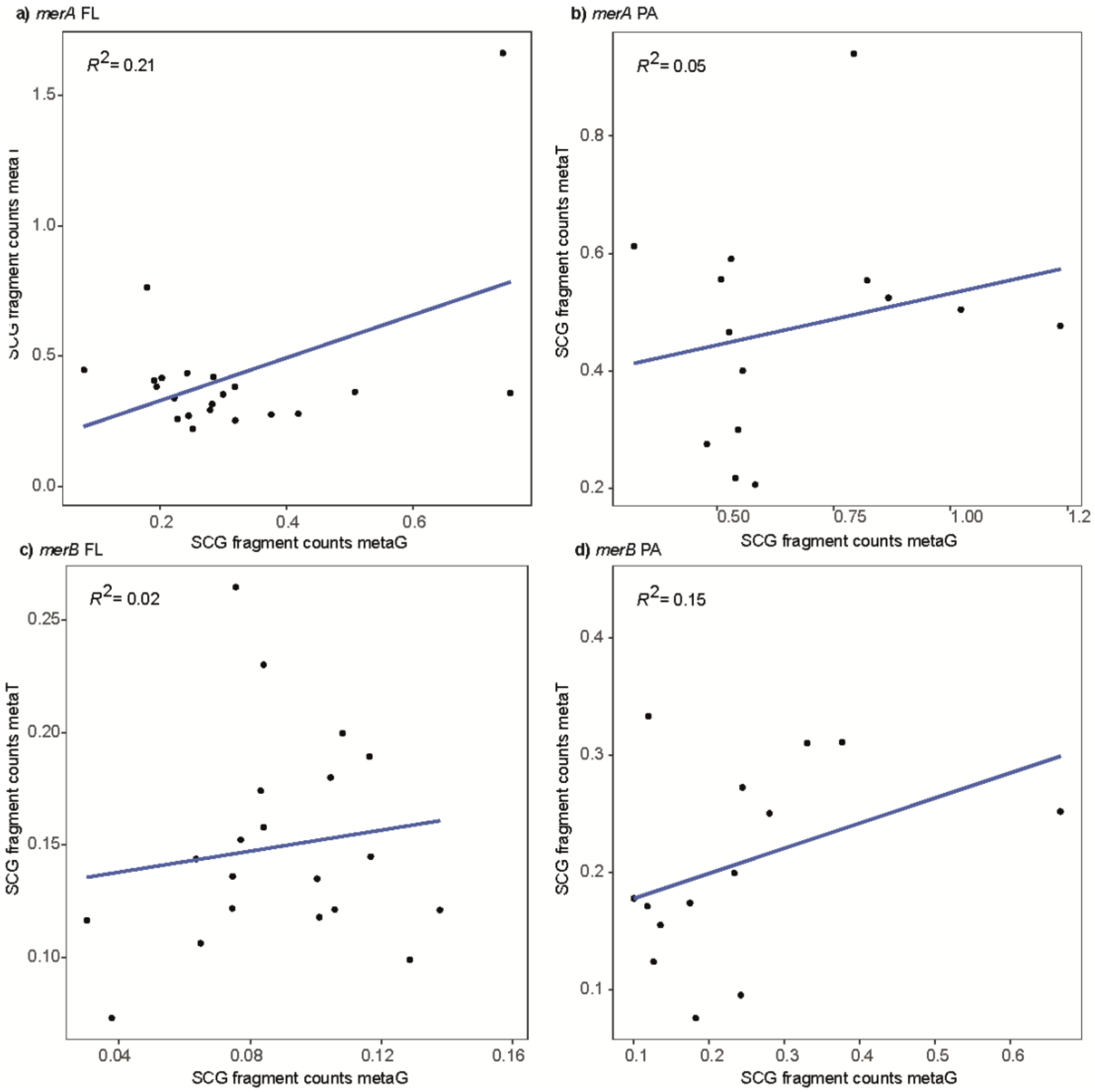

Fig. S3. Gene abundance (metaG) versus expression (metaT). Relative abundance normalized by single copy genes fragment counts of *merA* **(a,b)** and *merB* **(c,d)** genes per lifestyle (FL: free-living; PA: particle attached). Square root transformation has been applied to all values to better visualize dispersion of the data. R^2 is indicates in each plot indicating no correlation between metaG and metaT in any of the genes per lifestyles.

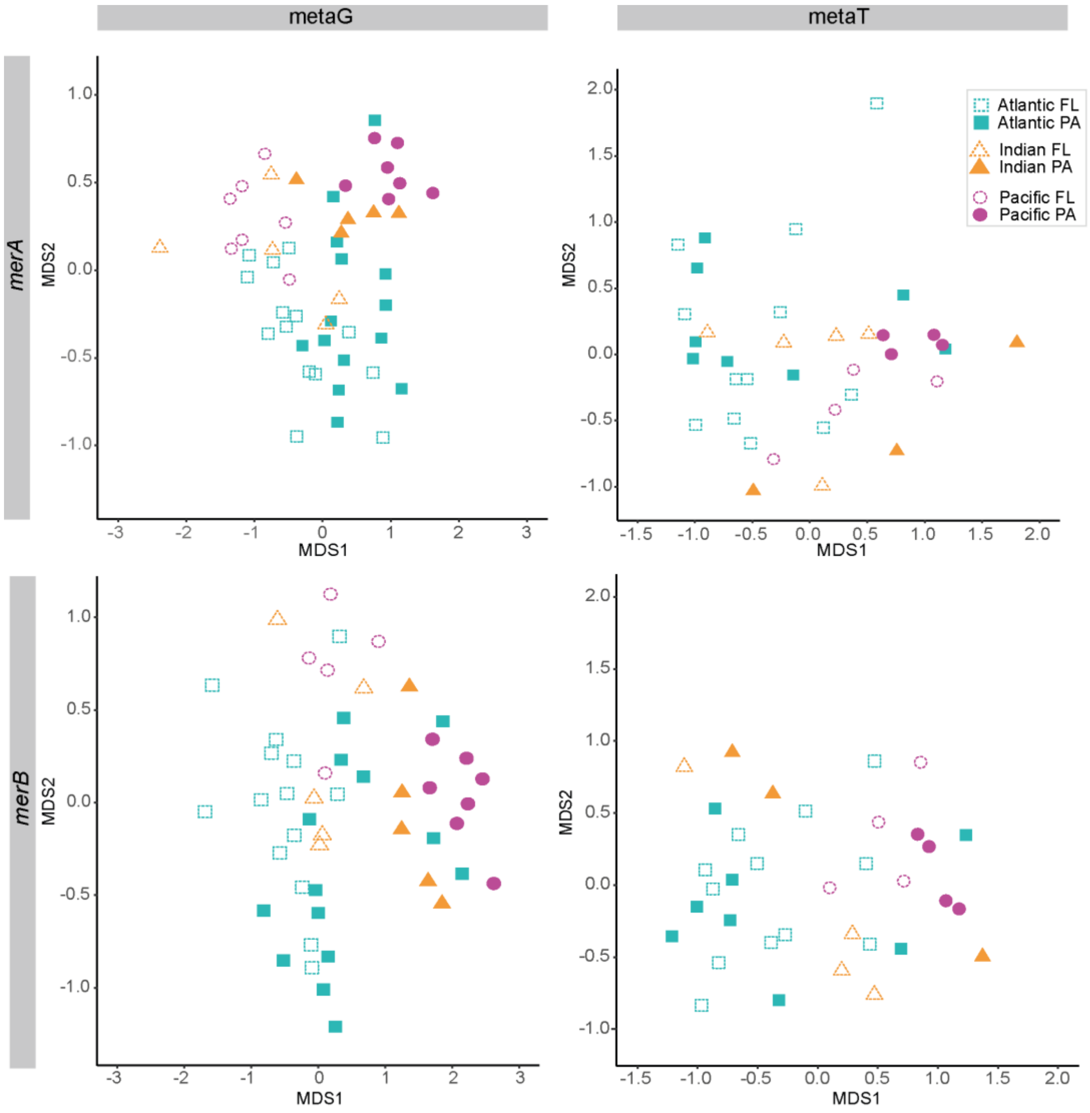

Fig. S4. **Non-metric multidimensional scaling of the Bray-Curtis distances between samples in metaG or metaT based on the analyses of the *merA* and *merB* genes.** Colored by ocean. Empty symbols with dashed lines indicate free-living (FL), and filled symbols indicate particle-attached (PA) bacterial communities.

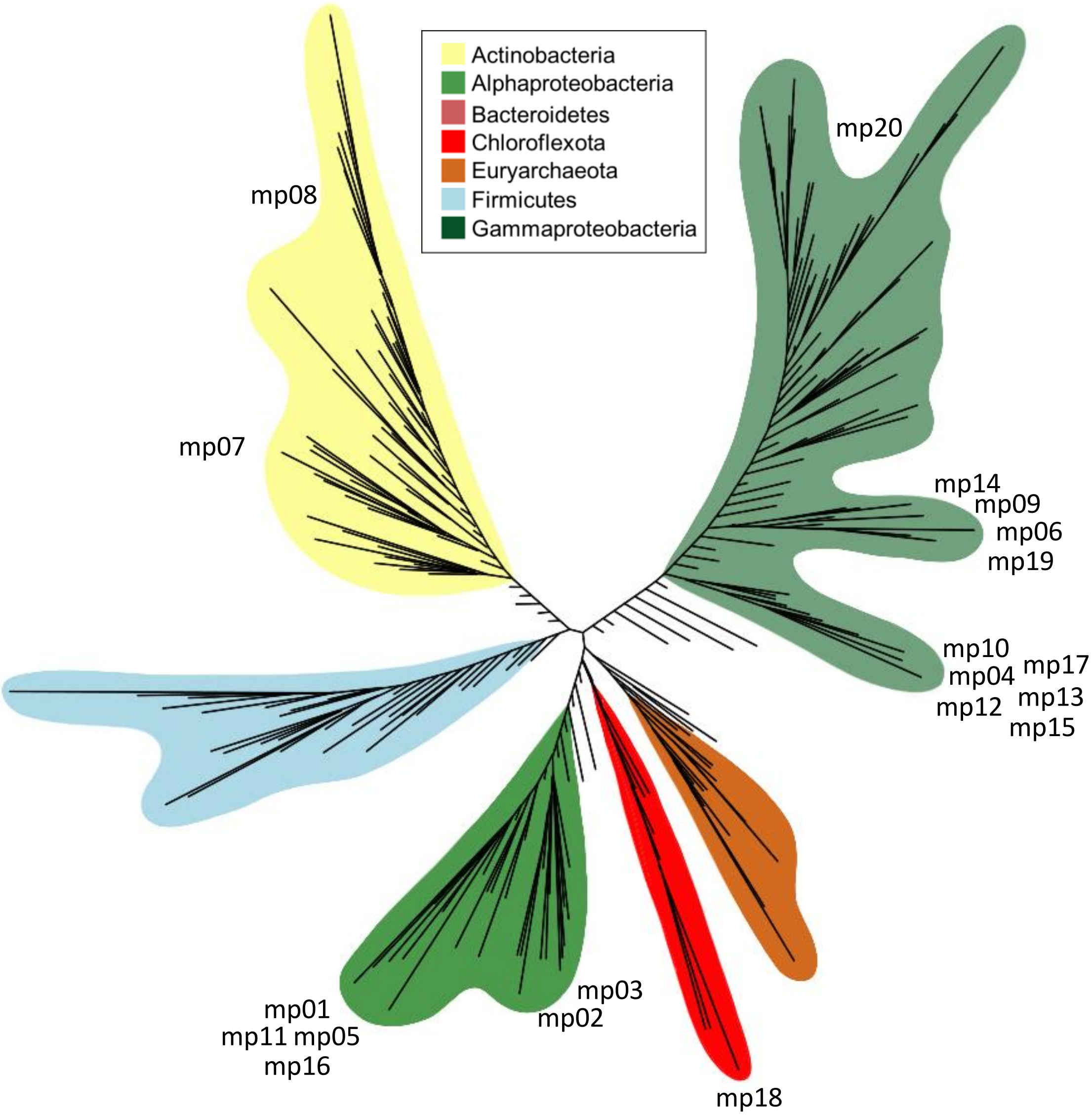

Fig. S5. MerA phylogenetic tree. Simplified unrooted phylogenetic tree of *merA* sequences obtained from the custom *mer* genes database created in the present study including the reconstructed phylogeny using sequences published in the Christakis et al. 2021 study [5] and the detected *merA* genes (mp01-mp20) from Malaspina metagenomes. Microbial groups with the highest diversity of *merA*+ microorganisms are denoted with colors.

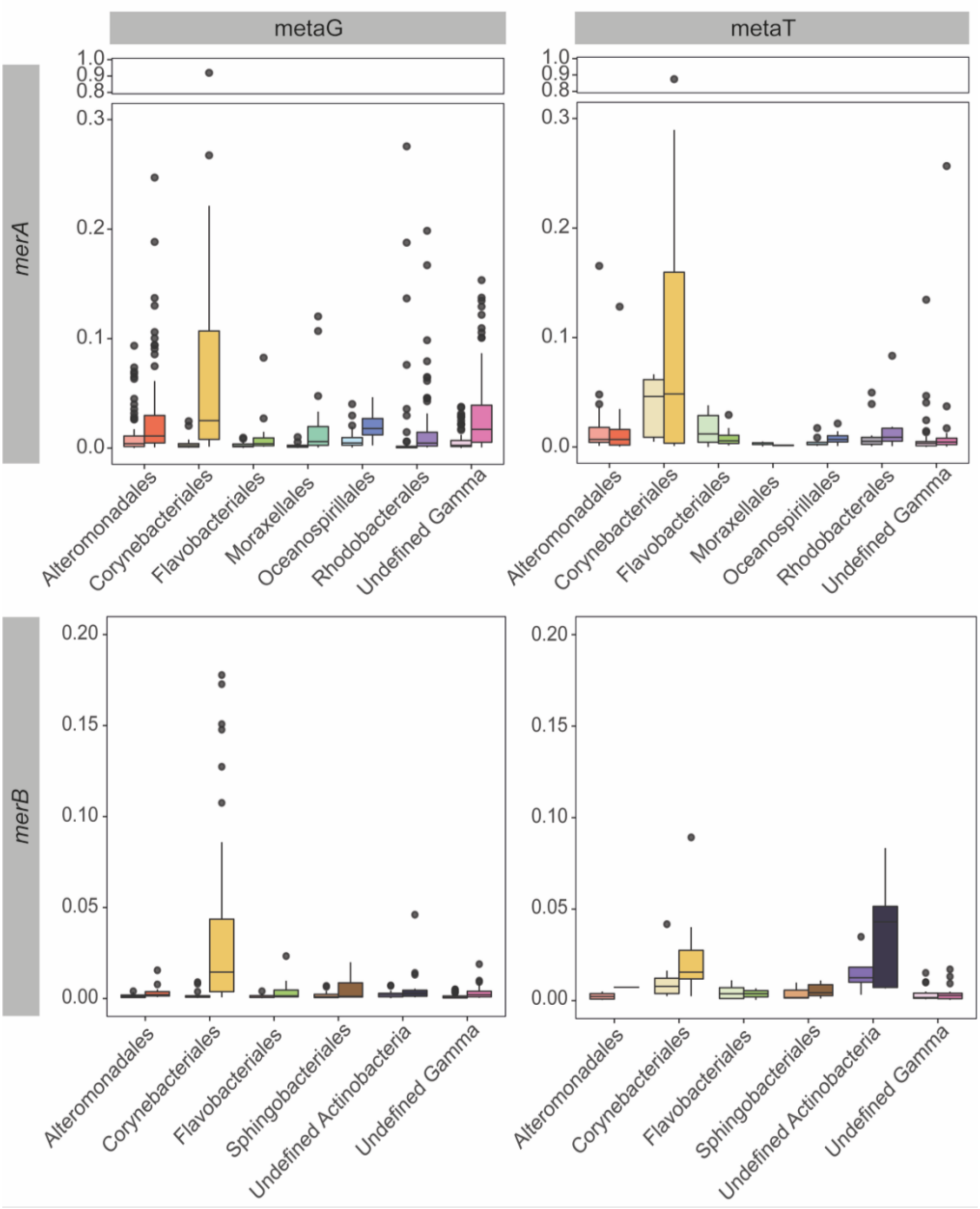

Fig. S6. **Relative abundance of *merA* and *merB* gene variants taxonomically classified at the order level in the metagenomes (abundance) and metatranscriptomes (expression).** The left boxplots indicate the relative abundance in the FL bacteria fraction (lighter colors) while the right boxplots the abundances in the PA communities (darker colours).

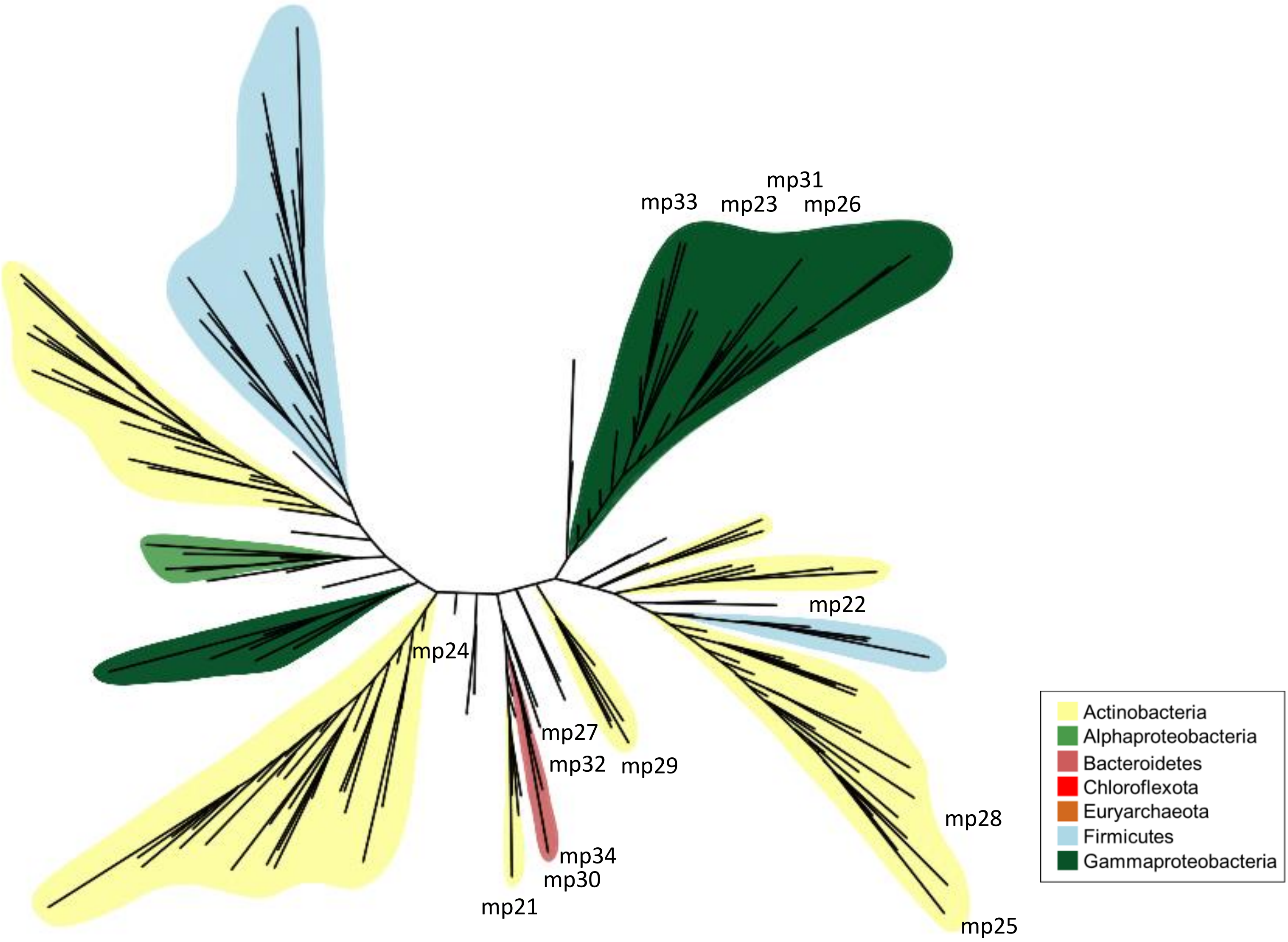

Fig. S7. MerB phylogenetic tree. Simplified unrooted phylogenetic tree of *merB* sequences obtained from the custom *mer* genes database created in the present study including the reconstructed phylogeny using sequences published in the Christakis et al. 2021 study [5] and the detected *merB* genes (mp21-mp34) from Malaspina metagenomes. Microbial groups with the highest diversity of *merB*+ microorganisms are denoted with colors.

Supplementary Tables

Table S1. Interesting environmental parameters used for elucidating possible drivers explaining abundance and expression of *merA* and *merB* genes across the global bathypelagic ocean.

Table S2. Abundance of *merA* and *merB* genes per station and size fraction (FL:free-living, PA: particle-attached). Values are the sum of all *merA* or *merB* gene variants counts normalized by single copy genes (SCG).

Table S3. Abundance of *merA* and *merB* genes per station and size fraction (FL:free-living, PA: particle-attached). Values are the gene counts normalized by single copy genes (SCG) of the different *merA* and *merB* gene variants detected separately.

Table S4. PERMANOVA results between Bray-Curtis dissimilarity matrix of *merA*/*merB* genes and Euclidian distance matrix of environmental variables. Asterisks indicate significance of the statistic test: * (p-value < 0.05), ** (p-value between 0.05-0.01), ***(p-value < 0.01).

Table S5. Taxonomic classification of *merA* and *merB* genes identified from global bathypelagic samples.

Table S6. Summary of the statistical analyses (Wilcoxon test) in order to elucidate if abundance (metaG) and expression (metaT) in the different taxonomic groups identified (at the order level) for *merA* and *merB* genes were different between free-living (FL) and particle-attached (PA) lifestyles. Significant differences are set to an alpha values of 0.05.

Table S7. *merA* and *merB* sequence variants abundance by means of normalized gene counts by single copy genes per each size fraction. FL: free-living. PA: particle-attached.
